# Knockdown resistance (*kdr*) gene of *Aedes aegypti* in Malaysia with the discovery of a novel regional specific point mutation A1007G

**DOI:** 10.1186/s13071-022-05192-z

**Published:** 2022-04-06

**Authors:** Mas Azlin M. Akhir, Mustafa F. F. Wajidi, Sébastien Lavoué, Ghows Azzam, Izhan Shahrin Jaafar, Noor Aslinda Ummi Awang Besar, Intan H. Ishak

**Affiliations:** 1 Insecticide Resistance Research Group (IRRG), School of Biological Sciences, Universiti Sains Malaysia, 11800 Minden, Penang Malaysia; 2 School of Distance Education, Universiti Sains Malaysia, 11800 Minden, Penang Malaysia; 3 Vector Control Research Unit, School of Biological Sciences, Universiti Sains Malaysia, 11800 Minden, Penang Malaysia; 4 Kota Bharu Public Health Laboratory, Kelantan State Health Department, 16010 Kota Bharu, Kelantan Malaysia; 5 Vector-Borne Disease Control Programme, Penang State Health Department, Anson Road, 10400 George Town, Penang Malaysia

**Keywords:** *Aedes aegypti*, *Kdr* resistance, A1007G, Pyrethroid, Insecticide resistance, Malaysia

## Abstract

**Background:**

Improved understanding of the molecular basis of insecticide resistance may yield new opportunities for control of relevant disease vectors. In this current study, we investigated the quantification responses for the phenotypic and genotypic resistance of *Aedes aegypti* populations from different states in Malaysia.

**Methods:**

We tested the insecticide susceptibility status of adult *Ae*. *aegypti* from populations of three states, Penang, Selangor and Kelantan (Peninsular Malaysia), against 0.25% permethrin and 0.25% pirimiphos-methyl using the World Health Organisation (WHO) adult bioassay method. Permethrin-resistant and -susceptible samples were then genotyped for domains II and III in the voltage-gated sodium channel (*vgsc*) gene using allele-specific polymerase chain reaction (AS-PCR) for the presence of any diagnostic single-nucleotide mutations. To validate AS-PCR results and to identify any possible additional point mutations, these two domains were sequenced.

**Results:**

The bioassays revealed that populations of *Ae*. *aegypti* from these three states were highly resistant towards 0.25% permethrin and 0.25% pirimiphos-methyl. Genotyping results showed that three knockdown (*kdr*) mutations (S989P, V1016G and F1534C) were associated with pyrethroid resistance within these populations. The presence of a novel mutation, the A1007G mutation, was also detected.

**Conclusions:**

This study revealed the high resistance level of Malaysian populations of *Ae. aegypti* to currently used insecticides. The resistance could be due to the widespread presence of four *kdr* mutations in the field and this could potentially impact the vector control programmes in Malaysia and alternative solutions should be sought.

**Graphical Abstract:**

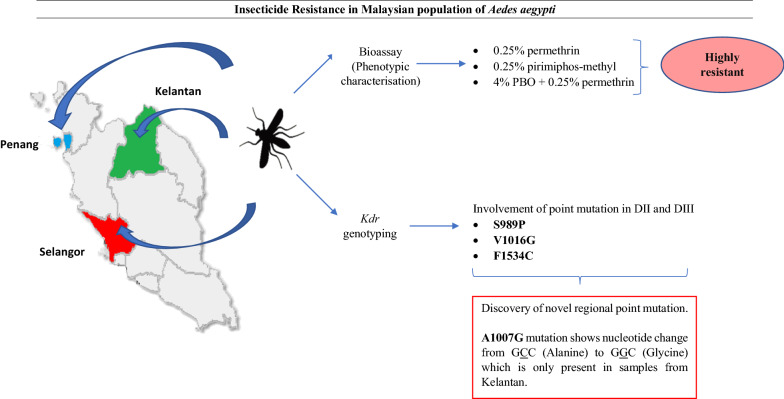

**Supplementary Information:**

The online version contains supplementary material available at 10.1186/s13071-022-05192-z.

## Background

The mosquito *Aedes aegypti,* is the primary vector for some arboviral diseases such as chikungunya [1], dengue [2], Zika [3, 4] and yellow fever [5], which have gained attention worldwide due to their fast-spreading trend [6–10]. This species is notoriously recognized in transmitting dengue fever and dengue haemorrhagic fever especially in tropical and sub-tropical countries due to their successful adaptation from feeding on animals in the natural forest ecosystems to preferentially feeding on humans in anthropogenic modified habitats [11, 12]. In Malaysia, dengue has become a major threat to public health with 93,344 cumulative dengue cases and 137 deaths reported in 2019, an increase of 81.8% and 69.1%, respectively, from the previous year [13–15]. Although the tetravalent dengue vaccine has recently been introduced and portrays a good efficacy profile during clinical trials, there are still some restrictions which needed to be overcome before large-scale commercialization [16].

Various approaches have been conducted to control population size and distribution of *Ae. aegypti*; most of them rely on the insecticide-based intervention targeting the immature and adult stages of this species [17]. In Malaysia, pyrethroids and organophosphates are routinely used during the vector control programmes conducted by the Ministry of Health, private pest control operators and local communities [18, 19]. Organophosphate and pyrethroid have different modes of action but both insecticides target the nervous system of the insect, which eventually leads to its death [20]. Such excessive use of these insecticide classes for a long period led to overdependence and improper usage of these insecticides eventually causing resistance within this vector.

Inappropriate usage and overexposure to insecticides that share the same mode of action can lead to the selection of several types of insecticide resistance in the mosquitoes: (1) the modification of the mosquito cuticle, leading to reduced penetration of the insecticide into the insect’s exoskeleton, (2) the presence of a single-nucleotide polymorphism (SNP) resulting in the modification of the nucleotide in the target gene and consequently changing the amino acid of the target sites in mosquito, (3) an increase in the enzymatic activity in detoxifying the insecticides or (4) changes in the mosquito behaviour enabling them to survive in the toxic environment [21].

The two resistance mechanisms commonly associated with pyrethroid resistance within insects are often associated with an increased metabolic detoxification activity [22] and insensitivity of the target sites such as the sodium channel gene, acetylcholinesterase (*Ace-1*) gene and gamma-aminobutyric acid (GABA) receptor [23]. In particular, several point mutations have been functionally identified in the *vgsc* gene that reduce its sensitivity by preventing the binding of the insecticide to the target gene, leading to the knockdown resistance (*kdr*) [24–27].

A total of 13 non-synonymous mutations have already been detected worldwide in the *vgsc* gene of pyrethroid-resistant populations of *Ae. aegypti*: G923V, L982W, I1011M and V1016G were first identified in 2003 [24]. In 2007, I1011V and V1016I mutations were reported from Latin America [25]. D1763Y mutation was observed in Taiwan in 2009 [28]. S989P and F1534C mutations were first reported from Thailand in the following year [29, 30]. In 2015, T1520I mutation was reported from India [31]. The first *kdr* mutation in domain I, V410L, was first observed in Brazil in 2017 [27]. A1007G mutation was first detected in Vietnam in the following year [32]. Recently, F1534L mutation has been reported in the pyrethroid-resistant Indian populations of *Ae*. *aegypti* [33]. Five of these 13 mutations (V410L, S989P, I1011M, V1016G and F1534C) have been functionally characterized in vitro in the expression system of *Xenopus* oocytes and confer *kdr* resistance in *Ae. aegypti* [24–29, 31, 34, 35].

In Malaysia, three common non-synonymous knockdown (*kdr*) mutations within the *vgsc* gene of *Ae. aegypti* are known to be associated with pyrethroid resistance. The mutations S989P, V1016G and F1534C correspond to the substitutions of the amino acid serine to proline, valine to glycine and phenylalanine to cysteine, respectively, within domains II and III. These mutations are widely established across Southeast Asia, not only across Malaysia [36–38], but also in Indonesia, Thailand, Singapore, Myanmar and Vietnam [39–42, 44].

Characterizing the target site resistance mechanism is an essential key to improve the management strategies of the vector control. Hence, the aim of this study is to further elucidate the insecticide resistance mechanism associated within Malaysian *Ae. aegypti* population phenotypically and genotypically.

## Methods

### Sampling and rearing of mosquitoes

Six populations of *Aedes aegypti* were collected in 2017 from three states in Malaysia, Penang, Selangor and Kelantan, by placing 100 ovitraps at residential areas for 5 days. The sites were selected based on the number of reported dengue cases in *idengue* website (https://idengue.mysa.gov.my/), which is the Malaysian national dengue database (Ministry of Health), and were routinely sprayed with insecticides, particularly permethrin and pirimiphos-methyl. Two sites were within each state (total = six sites): Sungai Dua (SD) and Balik Pulau (BP) in Penang, Alam Budiman (AB) and TUDM, Subang (TDM) in Selangor, and Pauh, Panji (PNJ) and Flat Buluh Kubu (FLT) in Kelantan. In each study site, ovitraps were set up at a distance between 5 to 10 m apart depending on the type of houses within that residential area. The ovitraps were randomly placed near potential breeding sources and were not exposed to direct sunlight. The traps were collected after 5 days and brought back to the insectary for culturing purposes.

The eggs of *Aedes* mosquitoes from all the localities were hatched, and upon emergence of first instar larvae, they were fed with larval food containing ground cat biscuit, beef liver powder, milk powder and yeast with a ratio of 2:1:1:1. The adult mosquitoes were morphologically identified to the species level based on the thorax pattern. The adult *Ae. aegypti* were supplied with 10% sucrose solution. All larvae and adults were maintained at 28 $\pm$ 2 $^\circ{\rm C}$ with a relative humidity of 75 $\pm$ 10%. The local susceptible laboratory strain obtained from the Vector Control Research Unit (VCRU) was used as a reference strain.

### WHO adult bioassay

The adult mosquito bioassay was performed according to the World Health Organisation (WHO) protocol [44]. The diagnostic dosage for *Aedes* mosquito was used for permethrin and the tentative dosage for *Anopheles* was used for pirimiphos-methyl. Twenty female 3–5-day-old *Ae. aegypti* were tested against 0.25% permethrin (Type I pyrethroid) and 0.25% pirimiphos-methyl (organophosphate) for 1 h, which was replicated five times. The susceptibility bioassay test was conducted at 28 $\pm$ 2 $^\circ{\rm C}$ with a relative humidity of 75 $\pm$ 10%. Subsequently, they were supplied with 10% sucrose solution ad libitum and maintained under insectary conditions. The numbers of dead and alive mosquitoes were recorded at 24 h post-exposure. After 24 h exposure, the whole bodies of the surviving mosquitoes were transferred and kept in − 80 $^\circ{\rm C}$ (for later quantification of metabolic resistance by real-time PCR) and those of the dead samples were preserved in silica gel inside microcentrifuge tubes for *kdr* genotyping.

### Synergist bioassay

The synergism assay was conducted as a first screening to investigate the potential role of a mixed function oxidase and esterase enzyme superfamily in the insecticide metabolic detoxification. Piperonyl butoxide (PBO) was used prior to permethrin exposure because it is known as a common synergist for pyrethroids [45]. It inhibits enzymes from the families of esterases and mixed function oxidases, which are mainly associated with metabolic resistance caused by pyrethroid, organophosphate and carbamate exposure. The test was performed on the individuals from all sampling sites because the percentage mortality was < 90% from the WHO bioassay test. The mortality of the individual mosquitoes was recorded after 24 h.

### Genotyping of *kdr* mutations in voltage-gated sodium channel (*vgsc*) gene in *Aedes aegypti *extraction of genomic DNA (gDNA)

The gDNA of 20 dead and alive samples from all localities were extracted from legs and wings following Livak protocol [46]. The DNA concentration and purity were measured using a Nanodrop spectrophotometer at 260 nm. The gDNA samples were stored in the − 20 $^\circ{\rm C}$ for the downstream application.

### Detection of the V1016G and F1534C mutations by using allele-specific PCR (AS-PCR) in *Aedes aegypti*

To determine the presence of V1016G and F1534C point mutations conferring pyrethroid resistance, 20 resistant and 20 susceptible individuals (from all localities and previously exposed to permethrin) were randomly genotyped using AS-PCR protocol (Additional file 1: Table S1) as previously described by Stenhouse et al. [40] and Yanola et al., [30].

Each reaction for the detection of V1016G mutation consisted of a total volume of 15 l$\mu$ of 1.25 mM MgCl_2_, 1 $\times$ PCR buffer (Thermo Scientific, USA), 0.3 M$\mu$ forward primer (Gly1016f), 0.2 M$\mu$ for each reverse primer (Gly1016r or Val1016r), 200 M$\mu$ dNTP mixture (Promega, USA), 1.25 units DreamTaq Hot Start DNA polymerase (Thermo Scientific, USA) and 25–100 ng of gDNA. The amplification was carried out using Bio-rad MyCycler™ Thermal Cycle (Hercules, CA, USA). The thermal cycling condition was: 94 $^\circ{\rm C}$ for 2 min (initial denaturation); 35 cycles at 94 $^\circ{\rm C}$ for 30 s (denaturation), 58 $^\circ{\rm C}$ for 30 s (annealing), 72 $^\circ{\rm C}$ for 30 s (extension) and 72 $^\circ{\rm C}$ for 2 min (final extension) [40].

For the amplification of F1534C in domain III segment 6 (DIII S6), we followed the AS-PCR protocol as described by Yanola et al. [30]. The PCR reaction was performed in a volume of 15 l$\mu$ with a final concentration of 0.5 mM MgCl_2_, 1.5 $\times$ PCR buffer (Thermo Scientific, USA), 0.3 M$\mu$ for each forward primer (F1534-f and C1534-f), 0.23 M$\mu$ reverse primer (CP-r), 0.2 mM dNTP mixture (Promega, USA), 1.25 units DreamTaq Hot Start DNA polymerase (Thermo Scientific, USA) and 25–100 ng of gDNA. The reaction was run at 95 $^\circ{\rm C}$ for 2 min (initial denaturation); 35 cycles of 95 $^\circ{\rm C}$ for 30 s (denaturation), 56 $^\circ{\rm C}$ for 30 s (annealing), 72 $^\circ{\rm C}$ for 45 s (extension) and 72 $^\circ{\rm C}$ for 2 min (final extension). The products were run in 3% agarose gel. The sizes of the two amplified fragments for V1016G mutation are 60 base pairs (bp) for valine and 80 bp for glycine. Meanwhile, the sizes of two amplified fragment for F1534C mutation are 93 bp for phenylalanine and 113 bp for cysteine.

### Validating polymorphic sites of the voltage-gated sodium channel (*vgsc*) gene in *Aedes aegypti* by DNA sequencing

Some of the AS-PCR products were sequenced to confirm the presence of V1016G and F1534C mutations and also to detect S989P mutation or any novel mutation. For that, the regions of DIIS6 and DIIIS6 in the *vgsc* gene (where the S989P, V1016G and F1534C mutations occur) were separately amplified by classic PCR using the protocol described in Stenhouse et al. [40] and Yanola et al. [30]. Each reaction was performed in 20 l$\mu$ final volume consisting of 1.5 mM MgCl_2,_ 0.2 M$\mu$ each for forward and reverse primers for DIIS6 and DIIIS6, 0.4 mM dNTP, 1 $\times$ PCR buffer and 1.0 units of GoTaq G2 Flexi DNA Polymerase (Promega, USA). PCR amplification began with 3 min at 95 $^\circ{\rm C}$ (heat activation step), 35 cycles at 95 $^\circ{\rm C}$ for 30 s (denaturation), 57 $^\circ{\rm C}$ for 30 s (annealing), 72 $^\circ{\rm C}$ for 30 s (extension) and 72 $^\circ{\rm C}$ for 3 min (final extension). PCR products were sent to Integrated DNA Technologies, Inc., for sequencing.

Chromatograms were edited using MEGA 6 Molecular Evolutionary Genetic Analysis software, version 6.06 [47]. All sequences determined in this study have been deposited in GenBank under accession number MT237357-MT237435. All nucleotide sequences of DIIS6 and DIIIS6 were then manually aligned (no indels needed) and we used the sequences of the pyrethroid-susceptible *Musca domestica* (GenBank accession number U38813.1) and *Ae. aegypti* China strain (GenBank accession number MF794972.1) to annotate them. Our phylogenetic matrix comprises 46 individuals and 491 and 346 nucleotide positions for domain II and domain III, respectively. We inferred the relationships among the individuals using a maximum parsimony method of phylogenetic reconstruction as implemented in PAUP* v4b10 [48]. The sequences of DIIS6 and DIIIS6 of the susceptible *Musca domestica* (GenBank accession number U38813.1) were selected as the outgroup to root the tree. A statistical parsimony network (method TCS) was built using the PopART [49, 50] software to determine the correspondence between haplotypes and the resistance/susceptible phenotypes.

### Statistical analysis

Percentage mortality 24 h post-exposure for each population was used to describe the phenotype status. The classifications of the susceptible and resistant phenotypes in the tested populations were interpreted according to the WHO guidelines [44]. The tested populations were susceptible if the percentage mortality ranged from 98 to 100%; mortality between 90 and 97% suggests that populations developed intermediate resistance; populations with mortality < 90% are considered resistant. Odds ratio (OR) and Fisher's exact test were conducted to compare the distribution of *kdr* genotypes between the alive and dead mosquitoes. Chi-square analysis was used to test the significant differences in percentage mortality with and without pre-exposure to PBO. All statistical analysis was performed using The Statistical Package for Social Science software (IBM SPSS Statistics version 24).

## Results

### WHO adult bioassay

**Fig. 1:**
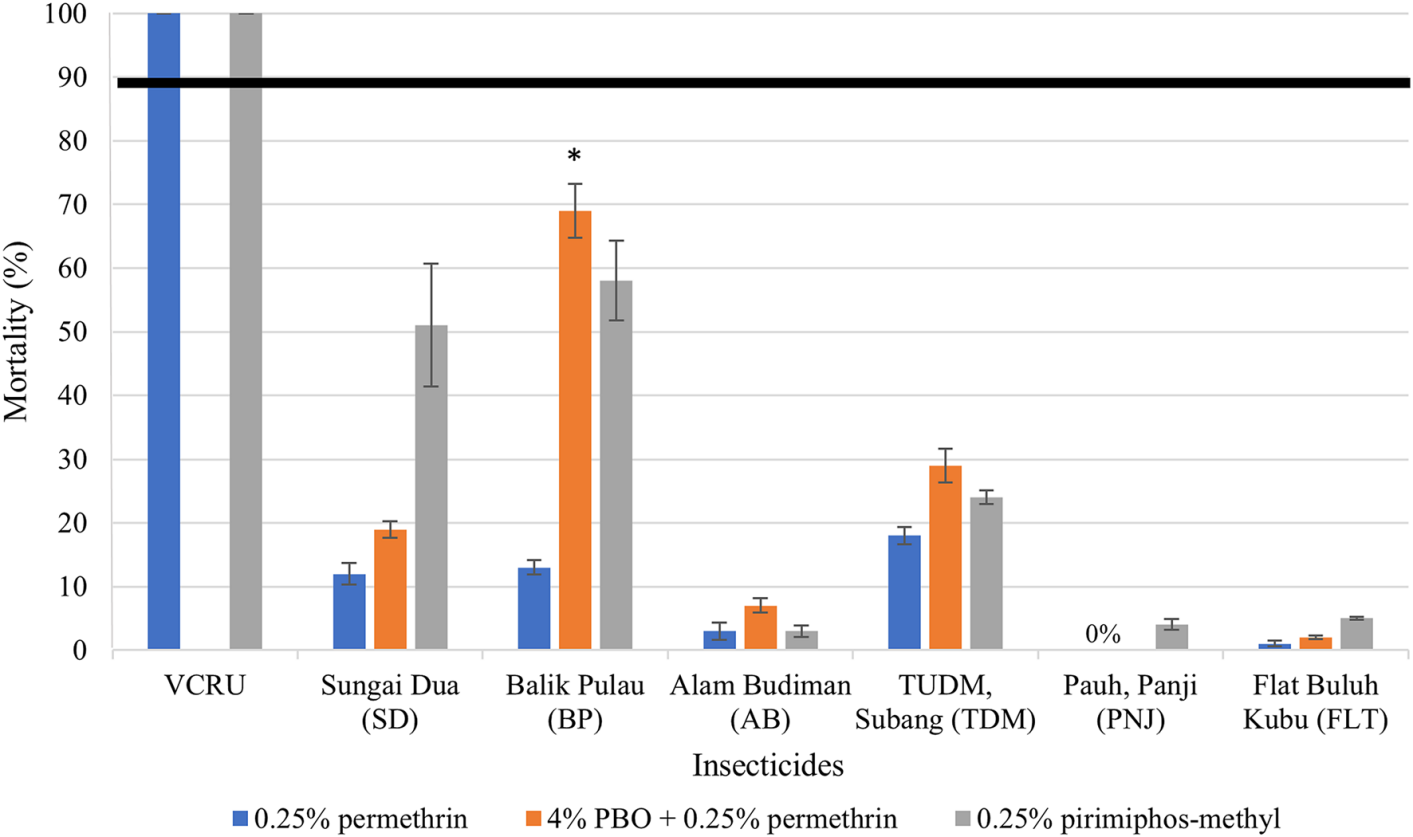
Percentage mortality of female *Aedes aegypti* from various locations across Malaysia toward two classes of insecticides and a synergist. Error bars represent 95% confidence interval (CI). Black horizontal line indicates resistant threshold level (mortality < 90% is considered phenotypically resistant; WHO, 2016). Statistically significant difference for the exposure with and without PBO is indicated by *P $<$ 0.05; 0% indicates no mosquitoes died 24 h post-exposure

WHO bioassays on the field strains revealed high phenotypic resistance in all six populations of *Ae. aegypti* from Selangor, Penang and Kelantan with percentage mortality < 90% after 24 h exposure toward permethrin and pirimiphos-methyl (Fig. 1). Percentage mortality varied from 0% to only 18% for 0.25% permethrin and from 3 to 58% for 0.25% pirimiphos-methyl. Full susceptibility was observed in VCRU laboratory strain *Ae. aegypti*, after the exposure to both insecticides (Fig. 1).

The lowest percentage of mortality toward 0.25% permethrin was observed in the populations from Pauh, Panji (PNJ) and Flat Buluh Kubu (FLT), Kelantan, with 0% and 1% mortality, respectively, indicating that these two populations developed very strong resistance and required additional assessment regarding the genetic mechanisms and distribution of such resistance. *Aedes aegypti* from TUDM, Subang (TDM), shows the highest mortality compared to other populations with 18%, although such percentage of mortality is still quite low. Resistance toward 0.25% pirimiphos-methyl was also observed in all populations with the highest resistance level recorded in Alam Budiman, Selangor (AB), with 3% mortality. Meanwhile, the population from Balik Pulau, Penang (BP), exhibited the highest mortality against this insecticide with 58% mortality.

### Synergism assay with piperonyl butoxide (PBO)

One-hour pre-exposure to 4% PBO before being exposed to permethrin increased the percentage mortality of all populations (except Pauh, Panji, PNJ) from 2 to 69% (Fig. 1). Pre-exposure to synergist provoked a large increase in mortality for populations from TUDM, Subang (TDM) (29%) and Balik Pulau, Penang (BP) (69%) compared to other populations. Furthermore, *Ae*. *aegypti* from Balik Pulau, Penang (BP), showed a significant increase in susceptibility toward permethrin (chi-square test, *X*^2^ = 7.244, *df* = 1, *P* < 0.05). No impact of pre-exposure to synergist PBO was observed in the population from Pauh, Panji (PNJ), with 0% mortality before and after exposure to the synergist.

### Detection of *kdr* mutation in the *vgsc* gene of Malaysian population of *Aedes aegypti*

One hundred sixty-seven samples including dead and alive mosquitoes from all populations were genotyped to determine the presence of S989P, V1016G and F1534C in DIIS6 and DIIIS6 and the allelic frequencies. Results of the genotyping are shown in Tables 1 (S989P), 2 (V1016G) and 3 (F1534C).

**Table 1:** Correlation of genotype S989P with the resistance phenotype toward permethrin for the field strain *Aedes aegypti*

States	Location	Phenotype status	*n*	S989P							
				Genotype			Alleles		Frequency P allele	Odd ratio (OR)	Fisher’s exact test (*P* value)
				S/S	S/P	P/P	TCC	CCC			
Penang	Sungai Dua (SD)	Resistant	3	0	2	1	2	4	0.667	0	0.333
		Susceptible	2	0	0	2	0	4	1		
	Balik Pulau (BP)	Resistant	4	0	0	4	0	8	1	$\infty$	0.165
		Susceptible	3	1	0	2	2	4	0.667		
Selangor	Alam Budiman (AB)	Resistant	4	2	2	0	6	2	0.25	$\infty$	0.424
		Susceptible	2	2	0	0	4	0	0		
	TUDM (TDM)	Resistant	3	3	0	0	6	0	0	0	0.571
		Susceptible	4	3	1	0	7	1	0.125		
Kelantan	Pauh, Panji (PNJ)	Resistant	10	10	0	0	20	0	0	N/A	0.999
		Susceptible	–	–	–	–	–	–	–		
	Flat Buluh Kubu (FLT)	Resistant	10	9	0	1	18	2	0.1	$\infty$	0.823
		Susceptible	1	1	0	0	2	0	0		

“–”: No individuals died during adult bioassay, thus the genotype and the allelic frequency cannot be determined

N/A: could not be determined because no individual died because of susceptible phenotype. $\infty$ suggests a high risk for the association between phenotype and genotype

To detect other possible point mutations in the *vgsc* gene of the permethrin-resistant Malaysian populations of *Ae. aegypti*, the DIIS6 and DIIIS6 regions of this gene were sequenced and aligned. The DIIS6 region contains codons 989, 1011 and 1016 whereas the DIIIS6 region contains the codon 1534. Overall, we detected six nucleotide substitutions; four of them are non-synonymous resulting in four amino acid differences between the permethrin resistant and susceptible samples.

Two of the non-synonymous substitutions were identified in DIIS6 at codons 989 and 1016. Samples from Sungai Dua (SD), Balik Pulau (BP), Alam Budiman (AB) and TUDM, Subang (TDM), were either heterozygous or homozygous for this double amino acid change. At codon position 989, the change of wild-type amino acid serine (TCC) to proline (CCC) is due to a T to C substitution at nucleotide position 52 (in our alignment). This mutation was frequently detected in populations from Penang, Selangor and one sample from Flat Buluh Kubu, Kelantan (FLT), with the allelic frequency ranging from 0.1 to 1.0 (Table 1).

A change from valine (GTA, wild type) to glycine (GGA) at codon position 1016 was detected within populations from Penang and Selangor (but not in those of Kelantan) with allelic frequency ranging from 5 to 55% (Table 2). For this point mutation, 167 samples were genotyped by AS-PCR and for 46 of them the DSII6 region was sequenced to confirm the AS-PCR’s results. However, a discrepancy was found for 18 samples which were first detected to be heterozygous (V/G) by AS-PCR but turned out to be homozygous wild type (V/V) after sequencing. This discrepancy may be the consequence of the presence of two consecutive alternative mutations in DIIS6 leading to AS-PCR genotyping error, hence resulting in false-positive results [36]. The amplification of the non-specific band happened because of the mismatch of the single base in the gene; hence, it is unable to prevent the non-specific amplification during the PCR extension [51].

**Table 2:** Results on the association of V1016G allele with the insecticide resistance phenotype

States	Location	Phenotype status	*n*	V1016G							
				Genotype			Alleles		Frequency G allele	Odd ratio (OR)	Fisher’s exact test (*P*-value)
				V/V	V/G	G/G	GTA	GGA			
Penang	Sungai Dua (SD)	Resistant	20	14	3	3	31	9	0.225	0.29	0.03
		Susceptible	12	2	8	2	12	12	0.5		
	Balik Pulau (BP)	Resistant	20	4	10	6	18	22	0.55	1.667	0.450
		Susceptible	13	5	5	3	15	11	0.538		
Selangor	Alam Budiman (AB)	Resistant	20	14	2	4	30	10	0.25	$\infty$	0.315
		Susceptible	3	3	0	0	6	0	0		
	TUDM (TDM)	Resistant	20	7	9	4	23	17	0.425	3.696	0.0234
		Susceptible	18	12	6	0	30	6	0.167		
Kelantan	Pauh, Panji (PNJ)	Resistant	20	10	10	0	30	10	0.25	N/A	0.999
		Susceptible	0	–	–	–	–	–	–		
	Flat Buluh Kubu (FLT)	Resistant	20	18	2	0	38	2	0.05	$\infty$	0.999
		Susceptible	1	1	0	0	2	0	0		

“–”: No individuals died during adult bioassay; thus, the genotype and the allelic frequency cannot be determined. N/A: it could not be determined because no individual died because of susceptible phenotype. $\infty$ suggests a high risk for the association between phenotype and genotype

**Fig. 2:**
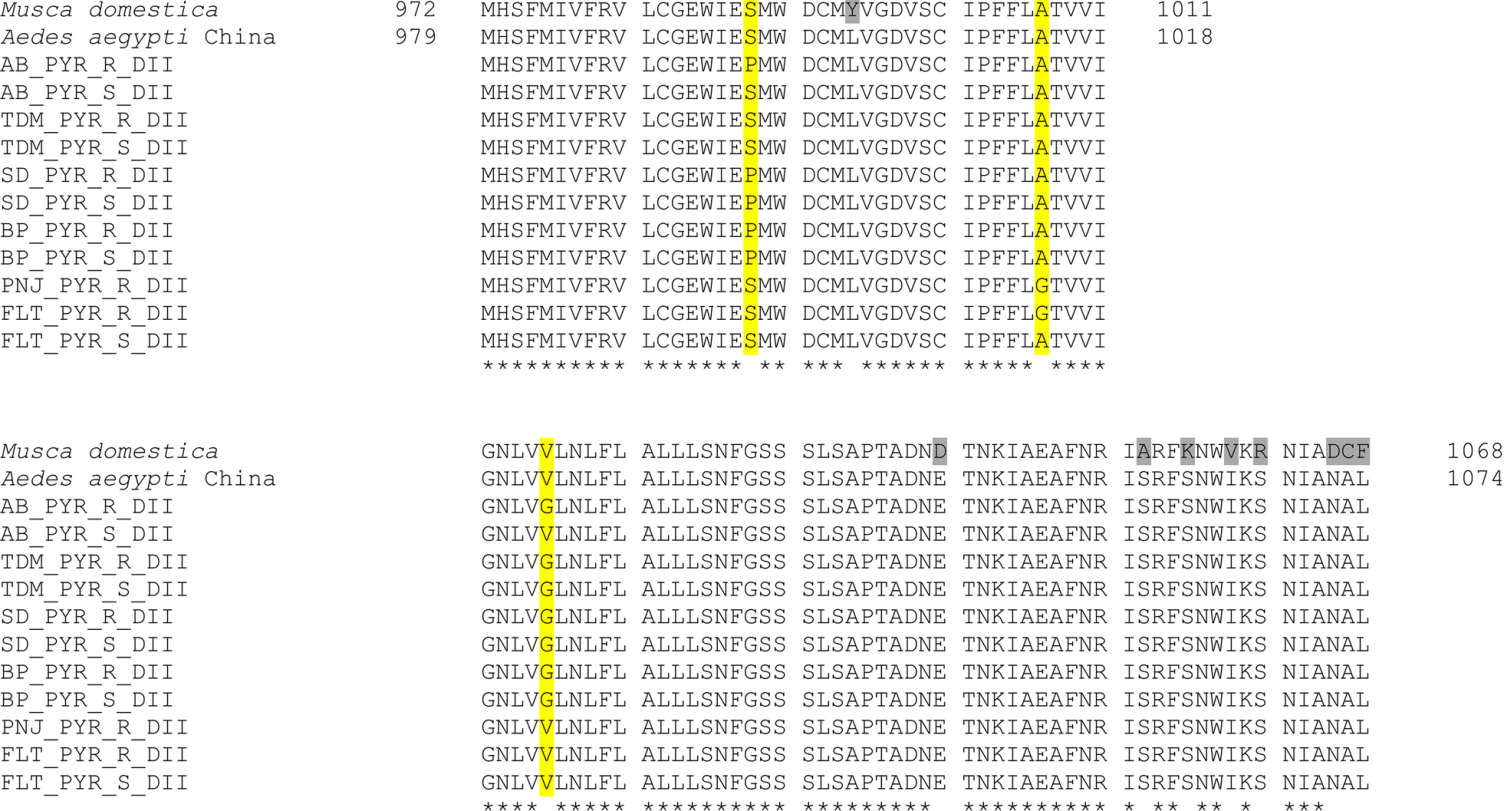
Alignment of the amino acid sequences for the partially amplified domain II, segment 6, of the voltage-gated sodium channel gene in the susceptible *Musca domestica* (GenBank accession number U38813.1) and *Aedes aegypti* from China (GenBank accession number AY663385.1) compared to the permethrin-resistant and permethrin-susceptible strains from six localities in Malaysia. The yellow highlighted letters show the positions of mutations S989P, A1007G and V1016G. Letters ‘R’ and ‘S’ in the sample name represent resistant and susceptible phenotypes, respectively. Asterisk (*) represents identical amino acid. Letters highlighted with grey are the conservative substitution

**Fig. 3:**
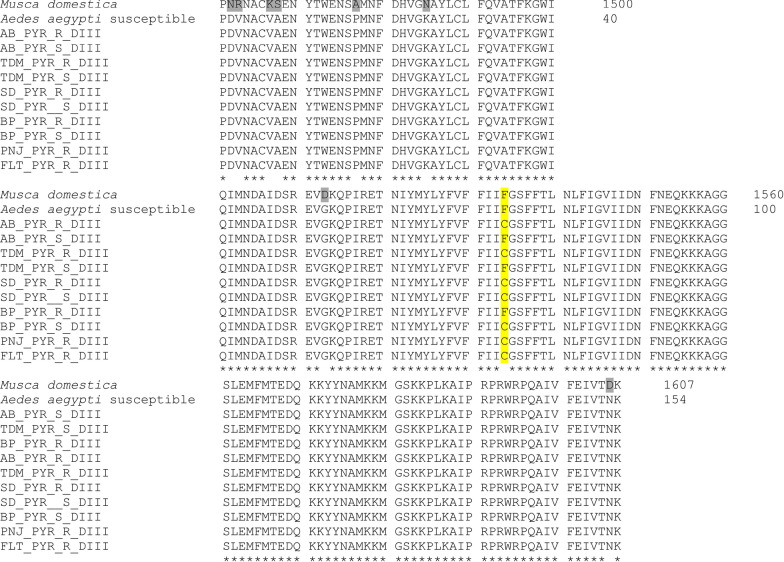
Amino acid alignment sequences of the partially amplified domain III, segment 6, of the voltage-gated sodium channel gene in the susceptible *Musca domestica* (GenBank accession number U38813.1) and *Aedes aegypti* (GenBank accession number MF794972.1) compared to the permethrin-resistant and -susceptible strains from six localities in Malaysia. Letters ‘R’ and ‘S’ represent resistant and susceptible phenotypes, respectively. The yellow highlighted letter shows the position of F1534C in domain III, segment 6. Asterisks (*) represent identical amino acid. Letters highlighted with grey are the conservative substitution

A novel non-synonymous substitution leading to a change at codon position 1007 from alanine (GCC) to glycine (GGC) was discovered. Only permethrin-resistant samples from Kelantan [Pauh, Panji (PNJ) and Flat Buluh Kubu (FLT)] have this novel amino acid substitution, with frequencies of 85% and 90%, respectively. Because this point mutation, 1007G, never co-occurs with other point mutations, whether S989P or V1016G in DIIS6, this mutation alone may be responsible in conferring the high resistance in the phenotypes (Fig. 2). However, direct sequencing reveals that 1007G always coexists with another point mutation in DIIIS6, the F1534C mutation (Figs. 2, 3). All samples from Kelantan (PNJ and FLT) were a mixture of heterozygous and homozygous for the double substitution mutations A1007G and F1534C (Additional file 2: Table S2).

In DIIIS6, a change from phenylalanine (TTC) to cysteine (TTG) at codon 1534 was detected in all six populations with allelic frequency ranging from 0.028 to 0.975 and populations from Kelantan showing the highest allele frequency for the permethrin-resistant samples, > 90%. Results showed that the mutant allele 1534C is common in the susceptible samples from Penang and TUDM, Subang (TDM) (Table 3, Additional file 3: Fig. S1).

**Table 3:** Results on the association of F1534C allele with the insecticide resistance phenotype

States	Location	Phenotype status	*n*	F1534C							
				Genotype			Alleles		Frequency C allele	Odd ratio (OR)	Fisher’s exact test (*P*-value)
				F/F	F/C	C/C	TTC	TGC			
Penang	Sungai Dua (SD)	Resistant	20	7	7	6	21	19	0.475	1.069	0.999
		Susceptible	12	3	7	2	13	11	0.458		
	Balik Pulau (BP)	Resistant	20	3	12	5	18	22	0.55	3.318	0.042
		Susceptible	13	8	3	2	19	7	0.269		
Selangor	Alam Budiman (AB)	Resistant	20	1	15	4	17	23	0.575	$\infty$	0.022
		Susceptible	3	3	0	0	6	0	0		
	TUDM (TDM)	Resistant	20	8	11	1	27	13	0.325	16.852	0.001
		Susceptible	18	17	1	0	35	1	0.028		
Kelantan	Pauh, Panji (PNJ)	Resistant	20	0	1	19	1	39	0.975	N/A	0.999
		Susceptible	0	–	–	–	–	–	–		
	Flat Buluh Kubu (FLT)	Resistant	20	1	1	18	3	37	0.925	$\infty$	0.02
		Susceptible	1	1	0	0	2	0	0		

“–”: No individuals died during adult bioassay; thus, the genotype and the allelic frequency cannot be determined. N/A indicates that it could not be determined because no individual died because of susceptible phenotype. $\infty$ suggests a high risk for the association between phenotype and genotype

### Association between *kdr* mutations at DIIS6 and DIIIS6 with pyrethroid resistance

To ascertain the impact of *kdr* mutations (S989P, A1007G, V1016G and F1534C) on pyrethroid resistance, association between each of these mutations and the permethrin-resistant phenotype was assessed by genotyping a total of 167 resistant and susceptible mosquitoes from all populations at DIIS6 and DIIIS6.

The S989P mutation in domain II is not significantly associated with the pyrethroid resistance in all populations (Fisher’s exact test, *P* = 0.146, OR = 0.512, 95% CI 0.188–1.391) and we presume the populations were not at Hardy-Weinberg equilibrium because of the small sample sizes tested for genotyping at codon 989 (Table 1). Fisher's exact test was conducted to compare the differences in V1016G of the allelic frequency between the resistant and susceptible phenotype from each locality. At codon 1016, the population from TUDM, Subang (TDM), exhibited significant association with permethrin resistance (Fisher’s exact test, *P* = 0.023, OR = 3.696, 95% CI 1.258–10.856) and the population from Sungai Dua, Penang (SD), was slightly correlated with permethrin resistance (Fisherʼs exact test, *P* = 0.030, OR = 0.290, 95% CI 0.097–10.864) (Table 2). In most of the localities, the differences in the allelic frequencies between alive and dead mosquitoes were not significantly correlated (Fisherʼs exact test, *P* = 0.429, OR = 0.922, 95% CI 0.549–1.550). More heterozygote mosquitoes survived after exposure to permethrin (Additional file 3: Fig. S1).

Statistical significance of the correlation between novel mutation A1007G with permethrin resistance in the population from Kelantan was not determined because of the low number of susceptible samples obtained after WHO bioassay. We noted, however, that mortality of the population from Kelantan was exceptionally low and the A1007G allele frequency in the resistant phenotype was very high ranging from 85 to 90% (Table 4).

**Table 4:** Association of potential novel mutation A1007G allele with the insecticide resistance phenotype

States	Location	Phenotype status	*n*	A1007G							
				Genotype			Alleles		Frequency G allele	Odd ratio (OR)	Fisher’s exact test (*P*-value)
				A/A	A/G	G/G	GCC	GGC			
Kelantan	Pauh, Panji (PNJ)	Resistant	10	0	3	7	3	17	0.85	N/A	0.999
		Susceptible	–	–	–	–	–	–	–		
	Flat Buluh Kubu (FLT)	Resistant	10	1	0	9	2	18	0.90	$\infty$	0.026
		Susceptible	1	1	0	0	2	0	0		

“–”: No individuals died during adult bioassay; thus, the genotype and allelic frequency cannot be determined. N/A: it could not be determined because no individual died because of susceptible phenotype. $\infty$ suggests a high risk for the association between phenotype and genotype

The frequency of the 1534C allele in domain III was significantly different between alive and dead mosquitoes from all six populations (chi-square test, *X*^2^ = 51.26, *df* = 1, *P* < 0.001). The 1534C mutant allele was highly significantly associated in the population from Balik Pulau (BP) and TUDM, Subang (TDM) (Fisher’s exact test, *P* = 0.042, OR = 3.318, 95% CI 0.5494–9.6451 and Fisher’s exact test, *P* = 0.001, OR = 16.852, 95% CI 2.073–136.932) (Table 3). No significant correlation was observed between 1534C genotype and permethrin resistance in Sungai Dua (SD) (Fisherʼs exact test, *P* = 0.999, OR = 1.069, 95% CI 0.387–12.95). From the Fisher’s exacts test, we found that Alam Budiman (AB), Pauh, Panji (PNJ) and Flat Bulu Kubu (FLT) were not in Hardy-Weinberg equilibrium, and we speculate that this might be due to the deficit of the heterozygote allele.

### Distribution of triple- and quadruple-loci of the genotypic combination in domain II and III of the *vgsc* gene in *Ae. aegypti*

Within the 46 samples of *Ae*. *aegypti* from Malaysia genotyped for DIIS6 and DIIIS6, we found 13 different combination patterns of substitutions with nine types of triple-locus and four types of quadruple-locus combinations observed. Most of the loci genotyped were a combination of two to three amino acid substitutions (compared to the wild-type combination S989 + A1007 + V1016 + F1534 [Type 10]). One to four different combinations of triple-locus genotype were detected in each population from Selangor and Penang. Two to three different combinations of quadruple-locus genotype were observed in the populations from Kelantan (Additional file 4: Table S3). Triple-locus wild-type homozygote, S989 + V1016 + F1534 (Type 1) and quadruple-locus wild-type homozygote, S989 + A1007 + V1016 + F1534 (Type 10) were found in four susceptible samples from Selangor and one susceptible sample from Kelantan, respectively. We noticed that triple-locus *kdr* genotypes (Types 3, 4, 5, 6) resulted in phenotypic resistance in the samples from Selangor and Penang. The presence of single-mutation S989 + 1016G + F1534 (Type 2) was only seen in the population from Selangor. Interestingly, the combination of the triple mutant 989P + 1016G + 1534C happened to be present in one susceptible sample from Penang. The individual which possessed this genotype combination survived after 24-h exposure period, suggesting the heterozygous allele at codon 1534 might have caused the lack of phenotypic resistance in this sample. Four combinations of quadruple-locus genotype were particularly present in the population from Kelantan with only three combinations of locus; 989P + A1007 + 1016G + F1534, S989 + 1007G + V1016 + 1534C and S989 + A1007/1007G + 1534C (Types 11, 12 and 13) were detected in permethrin-resistant samples and one combination, S989 + A1007 + V1016 + F1534 (Type 10), was found only in the permethrin-susceptible sample. Those loci consist of the new mutation with a high number resulting in 15 individuals (Kelantan population) surviving after the exposure to permethrin.

### Haplotype distribution and polymorphism analysis of the *vgsc* gene fragment in Malaysian populations of *Ae. aegypti*

**Fig. 4:**
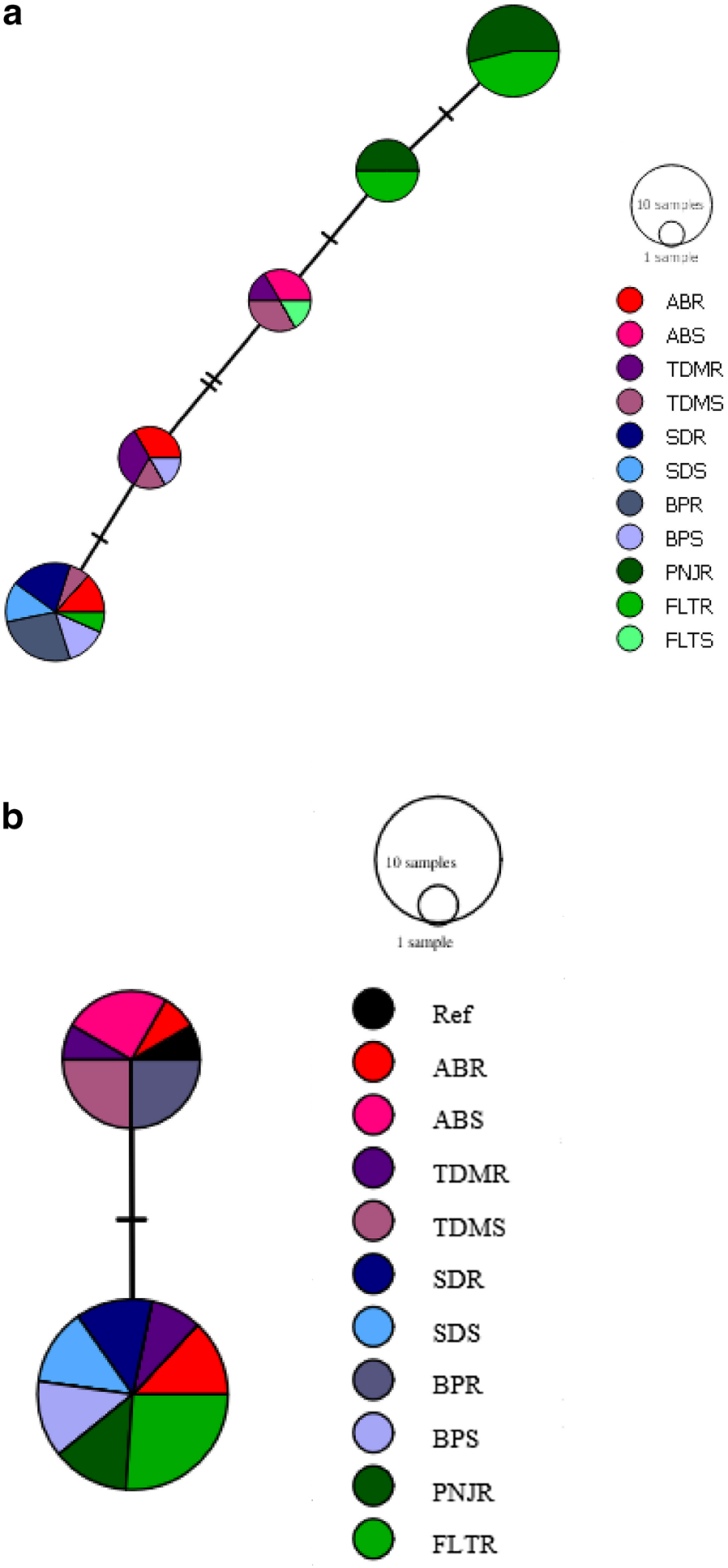
TCS network for the *vgsc* haplotype of fragment domain II (**a**) and III (**b**) between permethrin-resistant and -susceptible samples in all six populations of *Aedes aegypti* in Malaysia. ABR: Alam Budiman resistant; ABS: Alam Budiman susceptible; TDMR, TUDM, Subang resistant; TDMS, TUDM, Subang susceptible; SDR: Sungai Dua resistant; SDS: Sungai Dua susceptible; BPR: Balik Pulau resistant; BPS: Balik Pulau susceptible; PNJR: Pauh, Panji resistant; FLTR: Flat Buluh Kubu resistant; FLTS: Flat Buluh Kubu susceptible

Seven haplotypes were identified with five haplotypes present in DIIS6 and two haplotypes observed in DIIIS6. These haplotype variations produced four amino acid substitutions. In the coding region of DIIS6, we found five polymorphic sites resulting in three non-synonymous changes (S989P, A1007G and V1016G mutations) and two synonymous changes. Meanwhile in the coding region of DIIIS6, one polymorphic site at codon 1534 could be observed leading to the two haplotypes created in this *vgsc* fragment. In general, the *vgsc* gene exhibits a low polymorphism level for all six populations in Malaysia with a low number of the mutational steps between the haplotypes in DIIS6 and III as shown in the TCS network (Fig. 4). From the TCS network analysis in DIIS6, four resistant haplotypes were observed with one singleton haplotype 4 (H4) and haplotype 5 (H5), which is a combination of the new mutation, and synonymous change was detected only in Kelantan samples.

Five different haplotypes with two major haplotypes have been established from all six *Ae. aegypti* populations in Malaysia (Fig. 4a). Interestingly, in haplotype 4 (H4) and haplotype 5 (H5), we found a novel regional mutation in DIIS6 segment 6, which comprised resistant samples from Kelantan state only and consisted of both the homozygous and heterozygous A1007G mutation. These samples from Pauh, Panji (PNJ) and Flat Bulu Kubu (FLT) were homozygous susceptible for S989P and V1016G mutations.

Only two haplotypes were found to be associated with the F1534C mutation in DIIIS6 of the *vgsc* gene. In DIIIS6, haplotype 1 (H1) was associated with F1534C mutation revealing that all resistant samples from six populations were homozygous resistant for F1534C mutation (Fig. 4b). Haplotype 2 (H2) has the homozygous susceptible allele, F1534.

There is no significant difference in the Tajima's D estimation in the *vgsc* fragment for either DIIS6 or DIIIS6, demonstrating a low number of the polymorphisms in the *vgsc* within those populations (Table 5). The presence of the predominant haplotypes in both DIIS6 and DIIIS6 suggested that there is selection pressure in the *vgsc* gene fragment in both DIIS6 and DIIIS6, which is in agreement with the existence of the *kdr* mutation in this Malaysian population of *Ae*. *aegypti*. Maximum parsimony phylogenetic tree analysis of the *vgsc* gene displayed an association between the pyrethroid resistance and the SNP of DIIS6 and DIIIS6 in the *vgsc* gene, respectively (Additional file 5: Fig. S2). The existence of polymorphism in exons 15 to 16 DIIS6 and exons 23 to 25 of DIIIS6 of *vgsc* gene potentially correlated with the permethrin resistance. Reconstruction of the maximum parsimony tree revealed the haplotype pattern within those domains clustered according to the phenotype of the mosquito samples.

**Table 5:** Summary statistics of the polymorphism at the *vgsc* fragment of domain II and III in the Malaysian population of *Ae. aegypti*

Domain	*N*	PS	*π*	*D*	Φ_ST_
DII	47	5	9.42961e+06	3.3123e+09^ns^	0.77635
DIII	34	1	0.0016929	1.3518^ns^	0.73996

*N*: number of samples; PS: number of polymorphic sites; $\pi$: nucleotide diversity; *D*: Tajima’s D (*P* < 0.05); Φ_ST_: AMOVA; ns: not significant

## Discussion

The worldwide development of insecticide resistance, which was already known a few decades ago, is a worrisome problem [10]. Malaysia is one of the countries confronted with this problem [52]. The present study examines the distribution and role of *kdr* site mutations in conferring pyrethroid resistance in the main dengue vector, *Ae. aegypti*, in Malaysia.

### Resistance profiles of *Aedes aegypti*

The dengue vector control programme highly relies on the use of chemical insecticides [53]. Application of chemical insecticides during thermal fogging and space spray to control adult *Ae*. *aegypti* has been conducted for several decades [18]. In the early 1970s, malathion was first used in fogging against adult mosquito vectors in Malaysia but its usage was discontinued and it was replaced by permethrin and deltamethrin in 1998 [52]. Currently, pyrethroid and organophosphate are the major classes of insecticides widely used to eliminate the *Aedes* mosquitoes during dengue outbreaks. These two classes of insecticides are simultaneously or successively used by the public health authorities [54] such as the Ministry of Health Malaysia throughout control programmes. The resulting high exposure of the natural populations of *Ae*. *aegypti* to these insecticides has led to strong selection on them with the development of resistance mechanisms against these insecticides [55]. The pest control industry uses the same classes of insecticides for the same purpose. Pyrethroid is also used in household insecticide products such as liquid vaporizers, aerosols, mosquito coils and mosquito mats [53]. These products have formulations containing active ingredients such as metofluthrin, tetramethrin, d-allethrin, transfluthrin, d-phenothrin, s-bioallethrin, cyphenothrin, deltamethrin, d-trans allethrin, prallethrin and permethrin [53, 54, 56]. The extensive usage of household insecticide products may exert selection pressure in the target mosquito population and thus might cause cross-resistance in these pyrethroid insecticides. High occurrence of permethrin resistance in *Ae*. *aegypti* is frequently reported in many countries from Southeast Asia including Singapore, Indonesia, Cambodia, Laos, Thailand, Myanmar and Vietnam [39, 42, 43, 57–62]. The increasing occurrence of pyrethroid resistance is alarming to governments and private sectors involved in vector control.

Apart from controlling adult *Ae*. *aegypti*, targeting at the larval stage using larvicidal treatment such as temephos also has been conducted as a part of vector control. Temephos has been widely used to control mosquito larvae since 1973. As a consequence, effective long-term usage of temephos has been compromised because of the development of resistance toward this particular insecticide [36, 63]. Therefore, surveillance of the phenotypic status of Malaysian *Ae*. *aegypti* across all the other states in Malaysia as well as their genotypic frequencies between the *kdr* alleles is crucial in the vector control programme and should be monitored over time. This could provide conclusive evidence for deciding which vector control strategies should be implemented to improve the management of the control programme.

Development of resistance against pyrethroid insecticide in Malaysia has been observed since 2001 and is gradually increasing yearly [52]. Insecticide rotation for permethrin and pirimiphos-methyl has been adopted by authorities during the control programme as one of the strategies in insecticide resistance management in Malaysia. However, the use of insecticides from the same class and having the same mode of action does not resolve the resistance problem. Furthermore, metabolic cross-resistance could occur between pyrethroid and organophosphate insecticides as a result of insecticide rotation [64]. Investigation is needed to confirm the involvement of metabolic cross-resistance in the Malaysian population of *Ae*. *aegypti*.

The overall low percentage mortality observed in the susceptibility bioassay in all six populations against 0.25% permethrin and 0.25% pirimiphos-methyl indicated that they are highly resistant against these insecticides, as shown recently [36–38]. This suggests that these insecticides are no longer effective for the control of these mosquito populations. Resistance ratio cannot be determined in this study because there were no knocked-down mosquitoes during the 1-h exposure to permethrin and pirimiphos-methyl because of their high resistance against both insecticides. Our results revealed that Penang populations of *Ae*. *aegypti* acquired resistance against pirimiphos-methyl in the last decade because, in 2011, a study showed that these populations were still susceptible to this insecticide [65]. Our results suggest that the current application of this insecticide during vector control programmes in Penang is now inefficient. Phenotypic pyrethroid resistance is also increasing in Malaysian populations of *Ae*. *aegypti*. It is important to note that we did not carry out intensity assays in addition to the 1 $\times$ diagnostic dose which was recommended in WHO guidelines [66] because of insufficient mosquito samples. Future studies such as dose-response study and intensity bioassay should be conducted in local populations to determine the strength of the resistance level among these populations, thus assisting in managing insecticide resistance. To understand the genetic basis of resistance mechanisms and their distribution in Malaysia, all six populations were subjected to additional investigation.

Resistance is usually the combination of two or more mechanisms. Pyrethroid resistance in *Ae*. *aegypti* is often associated with target site resistance and metabolic resistance [22, 23]. A few studies conducted in Malaysia reveal that several non-synonymous mutations (S989P, V1016G and F1534C) are present in the *kdr* gene of *Ae*. *aegypti*. None of them are reported in *Ae*. *albopictus*. The first report on the occurrence of point mutations in the *kdr* gene in a Malaysian population of *Ae*. *aegypti* was published in 2010. The authors found V1016G and F1534C mutations associated with the pyrethroid resistance in *Ae*. *aegypti* populations from Penang, Kuala Lumpur, Kota Bharu and Johor Bharu [36]. We also observed a significant correlation between these mutations in several populations in Malaysia, indicating that the combination of these two mutations increases resistance against pyrethroid type II. In 2018, Rasli et al. [37] reported the co-occurrence of S989P mutation with the previously reported V1016G mutation in permethrin-resistant wild populations from Kedah and Johor. Subsequently, Leong et al. [38] found the presence of those mutations in the populations from Selangor associated with pyrethroid and DDT resistance.

The wide spread of these point mutations of S989P, V1016G and F1534C in the *kdr* gene of the Malaysian populations *Ae*. *aegypti* might explain the large-scale usage of the insecticide from the pyrethroid class, which had been extensively used by the government and the private sector to control the population of mosquito vectors and especially *Aedes* species in Malaysia.

### A novel mutation in the Malaysian *Aedes aegypti* population

We discovered a novel substitution mutation, A1007G, from the Kelantan population (with a frequency $\ge$ 85%), which occurs in DIIS6 without the association of S989P and V1016G mutations. A1007G, however, co-occurs with the mutation F1534C in DIIIS6. We hypothesize that A1007G has a similar function as the other *kdr* mutations since this mutation is located within one of the four specific amino acid residues in the P-region segments, DIIS6 [67]. The change of nucleotide C to G at position 1007 in DIIS6, leading to the amino acid substitution from alanine to glycine, presumably gives rise to the alteration of the target site in the sodium channel, hence reducing its sensitivity toward permethrin. This is further justified by the results from a synergist assay that showed PBO might be considered a partially ineffective synergist. Modification in the insect voltage-gated sodium channel, which normally regulates sodium ions within the gene, makes the channel less functional, hence delaying the closing of the channel [68, 69].

Direct neurophysiological analysis has not yet been done. This step will be crucial in seeking evidence to test our hypothesis that the mutation A1007G is directly responsible for causing pyrethroid resistance within the permethrin-resistant population. Our study also has a limitation in validating this new mutation. Due to facility constraints, we were unable to conduct the functional validation of our potential novel substitution mutation, A1007G, expressed in *Xenopus* oocytes. By conducting the experiment, we might possibly learn the conformational occurrence of this new mutation in conferring pyrethroid resistance. However, high occurrence of the 1007G allele frequency together with the 1534C allele in that population provides us information showing that the co-occurrence of this new mutation with F1534C could be one of the factors contributing to pyrethroid resistance in the Kelantan population.

Other regional mutations in the Asian continent were also previously detected. Point mutation L982W in DIIS6 is associated with the DDT and pyrethroid cross resistance in the resistant population of *Ae*. *aegypti* in Vietnam [24]. In 2009 in Taiwan, the association of a novel mutation D1763Y with the mutation V1016G conferred high-fold resistance to permethrin in local populations [28]. These coexisting mutations likely cause a synergistic effect in knockdown resistance toward permethrin, reducing the sensitivity of the *vgsc* gene toward permethrin by 190-fold. The presence of the T1520I and F1534L mutations in the DIIIS6 of the *kdr* gene in the Indian *Ae*. *aegypti* population might be partly responsible for the pyrethroid resistance as well [31, 33]. These new mutations have yet to been functionally confirmed in the oocytes' system.

Outside Malaysia, Lien et al. [32] discovered this new mutation (A1007G) in pyrethroid-resistant populations from Vietnam. As far as we know, all other studies were unable to detect this mutation in other countries (see for example [70]), suggesting that A1007G has a geographically limited distribution, similar to local mutations V410L [35], G923V, L982W [24], I1011V/M [25], T1520I [31], F1534L [33] and D1763Y [28].

High frequency of the *kdr* mutations S989P, A1007G, V1016G and F1534C was detected amongst resistant samples of the six populations in Malaysia, demonstrating target site resistance is associated with resistance to permethrin. The low genetic diversity of the *vgsc* gene fragment spanning these mutations indicates that this gene is under selection pressure and supports the hypothesis that knockdown resistance plays a role in the pyrethroid resistance within Malaysian population of *Ae*. *aegypti*. This pattern was also observed by Ishak et al. [36], stating that F1534C mutation is under selection pressure across Malaysia, thus resulting in the reduction of genetic diversity within DIIIS6 of the *vgsc* gene. However, this scenario was not detected in the Malaysian population of *Ae. albopictus* since to date there has been no further attempt to elucidate the genetic diversity in the pyrethroid-resistant strain*.* In China, Zhou et al. [71] reported alleles 1016G and 1532T might have evolved from the susceptible *Ae*. *albopictus*. In the present study, we found two *kdr* mutations commonly classified under the same haplotype within domain II of the *vgsc* gene. This is an alarming observation that could affect the control of *Ae*. *aegypti* in Malaysia because of survival adaptation of the target mosquitoes to the same class of insecticide used in the control programme.

### Role of metabolic resistance in *Aedes aegypti*

A major increase of the susceptibility in Balik Pulau, Penang (BP), and a slight increase of the susceptibility in the other locations after pre-exposure to PBO suggest that metabolic resistance could be involved in conferring permethrin resistance in the Malaysian population of *Ae*. *aegypti*. PBO is widely known as the most frequent synergist used with the combination of the pyrethroid insecticide in controlling the resistant mosquitoes [72]. In general, synergists such as PBO can act as enzyme inhibitors in the metabolic enzyme defence system and act by binding the PBO metabolites to the enzyme from a superfamily group of monooxygenases P45O and non-specific esterases, hence resulting in the detoxification of enzymes to oxidize. Thus, the effectiveness of the pyrethroid will increase against pyrethroid resistant mosquitoes [45]. In the present study, pre-exposure to PBO might be considered an ineffective synergist against resistant *Ae*. *aegypti* mosquitoes as it is unable to restore full susceptibility after exposure toward permethrin. To note, no mortality was observed in the population from Pauh, Panji (Kelantan), after pre-exposure to PBO, suggesting that target site resistance might play a major role in this population. There might be other possible involvement of the resistance mechanism such as modification of the mosquitoes' cuticle within the Malaysian population, as the enhancement of the metabolic enzyme system can lead to reduced penetration of insecticides in the cuticular insects [73, 74].

## Conclusions

By elucidating the resistance mechanism involved in the Malaysian strain of *Aedes aegypti*, we can determine the geographical distribution of the mutations involved along with their frequencies. The present study shows the high occurrence of the reported mutations and detects new point mutations in the *vgsc* gene within the Malaysian permethrin-resistant strain. Henceforth, surveillance and monitoring of these mutations in the *vgsc* gene should be conducted regularly to detect any possible involvement of new point mutations and the frequency level. This can provide insightful decision-making hints toward the proper usage of insecticides against the target vector and to control the level of resistance within the target mosquito population in Malaysia. Understanding the resistance mechanism involved in the mosquito population will help the authorities to better plan vector control programmes.

## Supplementary Information

**Additional file 1: Table S1.** Sequence of oligonucleotides used to amplify the specific regions of the voltage-gated sodium channel (*vgsc*) gene in *Aedes aegypti.*

**Additional file 2: Table S2.** Additive effect of the *vgsc* gene fragment at domain II and III in *Ae*. *aegypti* population collected from Pauh, Panji (PNJ), and Flat Buluh Kubu (FLT), Kelantan.

**Additional file 3: Fig. S1.** Illustration map of Malaysia; three states in Malaysia where the resistance study was conducted and the distribution of *kdr* allele at codon 989, 1016 and 1534 in the phenotype resistant and susceptible field population of Malaysian *Ae*. *aegypti*.

**Additional file 4: Table S3.** Triple loci and quadruple loci of *kdr* genotype combination (S989P, A1007G, V1016G and F1534C) with pyrethroid resistance in *Ae. aegypti*.

**Additional file 5: Fig. S2.** Maximum parsimony phylogenetic tree of the *vgsc* fragment spanning exon 15 to exon 16 in domain II (a) and exon 23 to exon 25 in domain III (b) shows a correlation between haplotype and phenotype of resistance and susceptible samples. Susceptible *Musca domestica* (GenBank accession number U38813.1) was used as an outgroup.

## Data Availability

All data generated or analysed during this study are included in this published article (and its supplementary information files). The sequences determined in this study have been deposited in GenBank (GenBank accession nos. MT237357-MT237435).

## Abbreviations

WHO: World Health Organisation
vgsc: Voltage-gated sodium channel
AS-PCR: Allele-specific polymerase chain reaction
SNP: Single-nucleotide polymorphism
Ace-1: Acetylcholinesterase
GABA: Gamma-aminobutyric acid
kdr: Knockdown resistance
VCRU: Vector Control Research Unit
PBO: Piperonyl butoxide
OR: Odds ratio
