# Supplementary material for: Knockdown resistance (kdr) gene of Aedes aegypti in Malaysia with the discovery of a novel regional specific point mutation A1007G
Source: Parasit Vectors. 2022 Apr 6;15:122. doi: 10.1186/s13071-022-05192-z (PMC8988349; doi:10.1186/s13071-022-05192-z)
Supplement: Supplementary file 1 — Additional file 1: Table S1. Sequence of oligonucleotides used to amplify the specific regions of the voltage-gated sodium channel (vgsc) gene in Aedes aegypti. [file 13071_2022_5192_MOESM1_ESM.docx]

| Method | Primer name | Primer sequence (5’-3’) | Product size | Region in sodium channel | Reference |
| --- | --- | --- | --- | --- | --- |
| AS-PCR | Gly1016f | ACCGACAAATTGTTTCCC |  | DIIS6 | Stenhouse et al., 2013 |
|  | Val1016r | GCGGGCAGCAAGGCTAAGAAAAGGTTAATTA | 60 bp |  |  |
|  | Gly1016r | GCGGGCAGGGCGGCGGGGGCGGGGCCAGCAAGGCTAAGAAAAGGTTAACTC | 80 bp |  |  |
|  | F1534-f | GCGGGCTCTACTTTGTGTTCTTCATCATATT | 93 bp | DIIIS6 | Yanola et al., 2011 |
|  | C1534-f | GCGGGCAGGGCGGCGGGGGCGGGGCCTCTACTTTGTGTTCTTCATCATGTG | 113 bp |  |  |
|  | CP-r | TCTGCTCGTTGAAGTTGTCGAT |  |  |  |
| Direct sequencing | IIP_F | GGTGGAACTTCACCGACTTC | 581 bp | DIIP-DIIS6 | Yanola et al., 2011 |
|  | IIS6_R | GGACGCAATCTGGCTTGTTA |  |  |  |
|  | IIIS6_F | GCTGTCGCACGAGATCATT | 635 bp | IIIS4-IIIS6 |  |
|  | IIIS6_R | GTTGAACCCGATGAACAACA |  |  |  |
