# Supplementary material for: Knockdown resistance (kdr) gene of Aedes aegypti in Malaysia with the discovery of a novel regional specific point mutation A1007G
Source: Parasit Vectors. 2022 Apr 6;15:122. doi: 10.1186/s13071-022-05192-z (PMC8988349; doi:10.1186/s13071-022-05192-z)
Supplement: Supplementary file 2 — Additional file 2: Table S2. Additive effect of the vgsc gene fragment at domain II and III in Ae. aegypti population collected from Pauh, Panji (PNJ), and Flat Buluh Kubu (FLT), Kelantan. [file 13071_2022_5192_MOESM2_ESM.docx]

| **Samples** | **Phenotype** | **Loci** | | | |
| --- | --- | --- | --- | --- | --- |
|  |  | **989** | **1007** | **1016** | **1534** |
| PNJ 1 | Resistant | S/S | A/**G** | V/V | **C**/**C** |
| PNJ 2 | Resistant | S/S | **G**/**G** | V/V | **C**/**C** |
| PNJ 3 | Resistant | S/S | **G**/**G** | V/V | **C**/**C** |
| PNJ 4 | Resistant | S/S | A/**G** | V/V | **C**/**C** |
| PNJ 5 | Resistant | S/S | G/**G** | V/V | **C**/**C** |
| PNJ 6 | Resistant | S/S | A/**G** | V/V | **C**/**C** |
| PNJ 7 | Resistant | S/S | **G**/**G** | V/V | **C**/**C** |
| PNJ 8 | Resistant | S/S | **G**/**G** | V/V | **C**/**C** |
| PNJ 9 | Resistant | S/S | **G**/**G** | V/V | **C**/**C** |
| PNJ 10 | Resistant | S/S | **G**/**G** | V/V | **C**/**C** |
| FLT 1 | Resistant | **P**/**P** | A/A | **G**/**G** | F/F |
| FLT 2 | Resistant | S/S | **G**/**G** | V/V | **C**/**C** |
| FLT 3 | Resistant | S/S | **G**/**G** | V/V | **C**/**C** |
| FLT 4 | Resistant | S/S | **G**/**G** | V/V | **C**/**C** |
| FLT 5 | Resistant | S/S | **G**/**G** | V/V | **C**/**C** |
| FLT 6 | Resistant | S/S | **G**/**G** | V/V | **C**/**C** |
| FLT 7 | Resistant | S/S | **G**/**G** | V/V | **C**/**C** |
| FLT 8 | Resistant | S/S | **G**/**G** | V/V | **C**/**C** |
| FLT 9 | Resistant | S/S | **G**/**G** | V/V | **C**/**C** |
| FLT 10 | Resistant | S/S | **G**/**G** | V/V | **C**/**C** |
| FLT 11 | Susceptible | S/S | A/A | V/V | F/F |

**Bold** letter shows a mutant amino acid.
