# Supplementary figures and images for: Knockdown resistance (kdr) gene of Aedes aegypti in Malaysia with the discovery of a novel regional specific point mutation A1007G
Source: Parasit Vectors. 2022 Apr 6;15:122. doi: 10.1186/s13071-022-05192-z (PMC8988349; doi:10.1186/s13071-022-05192-z)

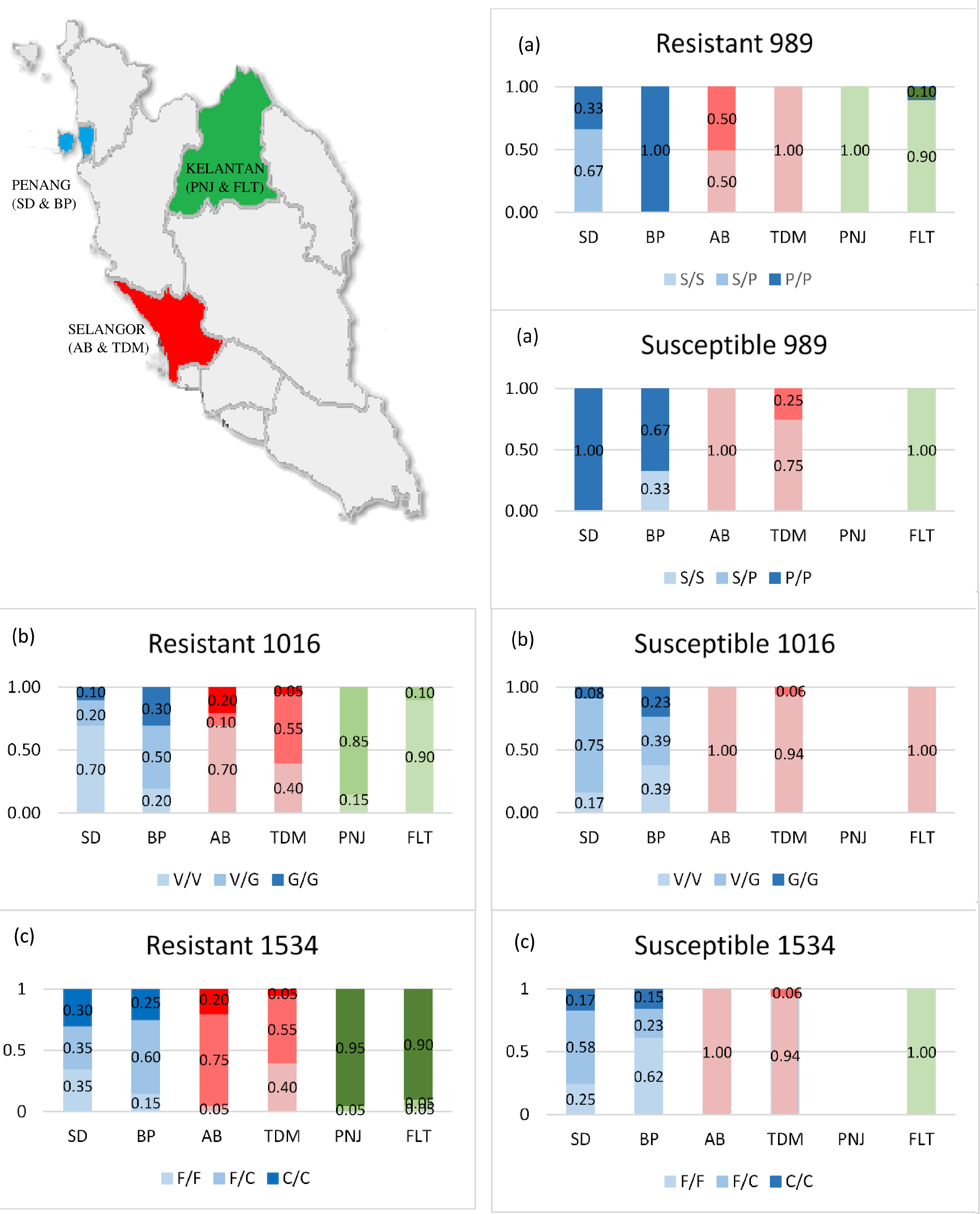

Supplement: Supplementary file 3 — Additional file 3: Fig. S1. Illustration map of Malaysia; three states in Malaysia where the resistance study was conducted and the distribution of kdr allele at codon 989, 1016 and 1534 in the phenotype resistant and susceptible field population of Malaysian Ae. aegypti. [file 13071_2022_5192_MOESM3_ESM.tif]

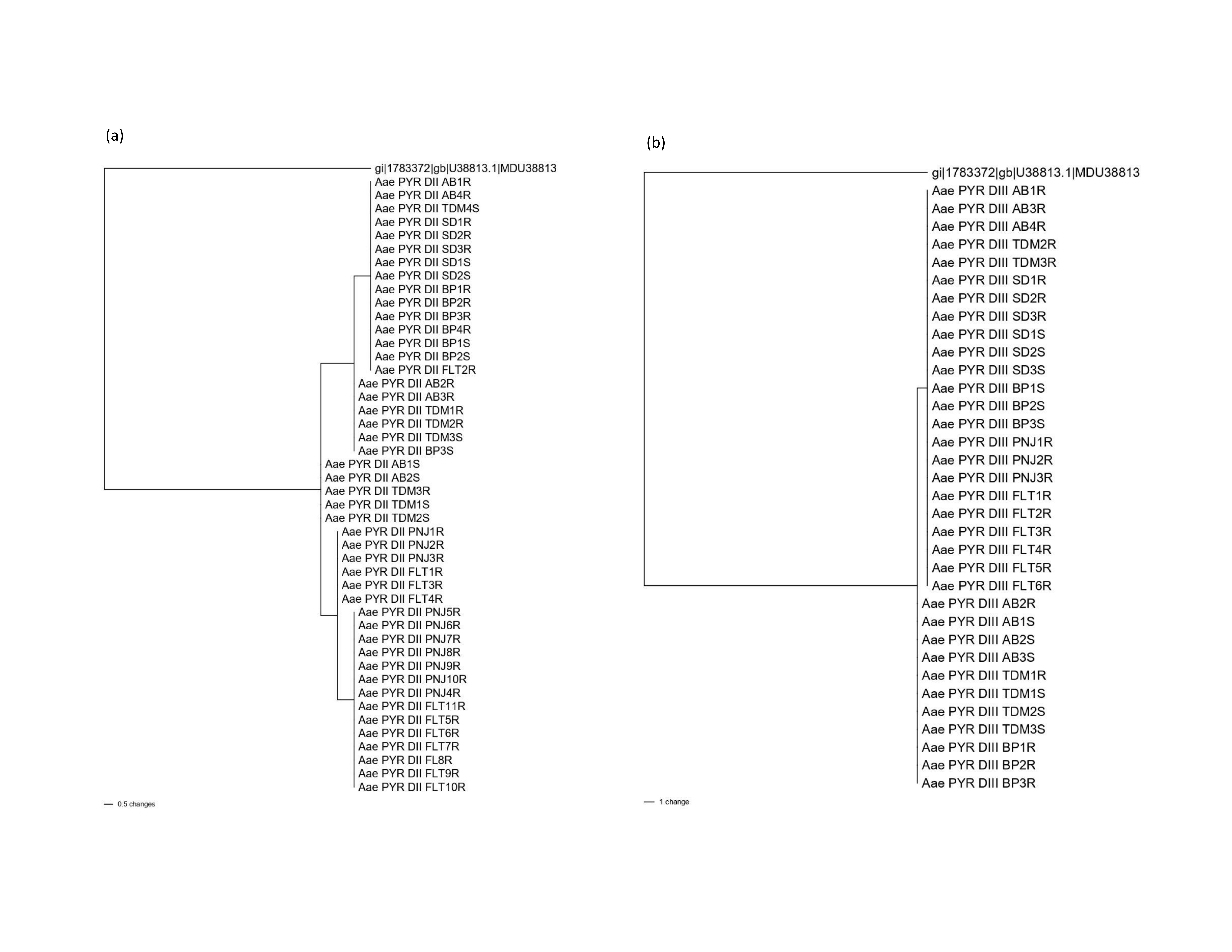

Supplement: Supplementary file 5 — Additional file 5: Fig. S2. Maximum parsimony phylogenetic tree of the vgsc fragment spanning exon 15 to exon 16 in domain II (a) and exon 23 to exon 25 in domain III (b) shows a correlation between haplotype and phenotype of resistance and susceptible samples. Susceptible Musca domestica (GenBank accession number U38813.1) was used as an outgroup. [file 13071_2022_5192_MOESM5_ESM.tif]
