# Supplementary material for: Knockdown resistance (kdr) gene of Aedes aegypti in Malaysia with the discovery of a novel regional specific point mutation A1007G
Source: Parasit Vectors. 2022 Apr 6;15:122. doi: 10.1186/s13071-022-05192-z (PMC8988349; doi:10.1186/s13071-022-05192-z)
Supplement: Supplementary file 4 — Additional file 4: Table S3. Triple loci and quadruple loci of kdr genotype combination (S989P, A1007G, V1016G and F1534C) with pyrethroid resistance in Ae. aegypti. [file 13071_2022_5192_MOESM4_ESM.docx]

| **Type** | **Genotype** | **Selangor** | | **Penang** | | **Kelantan** | | **Combined** | | **Mortality** |
| --- | --- | --- | --- | --- | --- | --- | --- | --- | --- | --- |
|  |  | **R** | **S** | **R** | **S** | **R** | **S** | **R** | **S** |  |
| 1 | SVF/SVF | 0 | 4 | 0 | 0 | 0 | 0 | 0 | 4 | 1.00 |
| 2 | S**G**F/S**G**F | 3 | 1 | 0 | 0 | 0 | 0 | 3 | 1 | 0.25 |
| 3 | S**G**F/S**GC** | 1 | 0 | 0 | 0 | 0 | 0 | 1 | 0 | 0.00 |
| 4 | S**GC**/S**GC** | 1 | 0 | 0 | 0 | 0 | 0 | 1 | 0 | 0.00 |
| 5 | S**G**F/**PG**F | 0 | 1 | 1 | 0 | 0 | 0 | 1 | 1 | 0.50 |
| 6 | S**G**F/**PGC** | 2 | 0 | 1 | 0 | 0 | 0 | 3 | 0 | 0.00 |
| 7 | **PG**F/**PG**F | 0 | 0 | 4 | 4 | 0 | 0 | 4 | 4 | 0.50 |
| 8 | **PG**F/**PGC** | 0 | 0 | 0 | 1 | 0 | 0 | 0 | 1 | 1.00 |
| 9 | **PGC**/**PGC** | 0 | 0 | 1 | 0 | 0 | 0 | 1 | 0 | 0.00 |
| 10 | SAVF/SAVF | 0 | 0 | 0 | 0 | 0 | 1 | 0 | 1 | 1.00 |
| 11 | **P**A**G**F/**P**A**G**F | 0 | 0 | 0 | 0 | 1 | 0 | 1 | 0 | 0.00 |
| 12 | S**G**V**C**/S**G**V**C** | 0 | 0 | 0 | 0 | 15 | 0 | 15 | 0 | 0.00 |
| 13 | SAV**C**/S**G**V**C** | 0 | 0 | 0 | 0 | 4 | 0 | 4 | 0 | 0.00 |

**Bold** alphabet represents a mutant amino acid.
